# Editorial: From safety to sense of safety

**DOI:** 10.3389/fpsyg.2025.1624676

**Published:** 2025-08-14

**Authors:** Anne Birgitta Pessi, Elizabeth Grant, Henrietta Grönlund, Birgitte S. Johansen, Maaret Kallio, Jenni Spännäri, Elisa Vähäsarja, Monica Worline

**Affiliations:** 1 Department of Practical Theology, Faculty of Theology, University of Helsinki, Helsinki, Finland; 2 Global Health Academy, Usher Institute University of Edinburgh, Edinburgh, United Kingdom; 3 Department of Cross-Cultural and Regional Studies, University of Copenhagen, København, Denmark; 4 Center for Positive Organizations, Faculty, Management and Organizations, Ross School of Business, University of Michigan, Ann Arbor, MI, United States

**Keywords:** safety, sense of safety, religious communities, organization, relationality, human relations, promoting sense of safety

## Sense of safety: universal core in and of humanity

Sense of safety is an essential foundation for human flourishing and wellbeing. It is a basic human need, grounded in the evolutionary history of human species as part of the ecosphere. Today, global challenges in social, political, and health spheres have undermined not only our safety and security but also our sense of safety. Sense of safety is an individual experience, but it is deeply rooted in social, communal, and societal contexts. Therefore, in research, we need to examine sense of safety with an understanding that broader societal conditions are significantly influenced by individual and grassroots-level experiences.

All individuals—regardless of ethnicity, age, or any other variable—need to feel safe. Sense of safety is a feeling of relative security—a comprehensive yet subjective psychological experience. It requires ongoing appraisal and is closely associated with a person's awareness and perception. In other words, sense of safety is related to actual safety but is never the same, and it is always about emotions. It is fundamentally social—never just about an individual—and should be studied in this context. Sense of safety is pivotal for individuals, communities, and societies. Sense of safety is often defined in research as a psychological phenomenon or an individual's internal feeling about safety, which is accompanied by a subjective perception of objective events (Zou and Meng, 2019, Nilsen et al., 2004). Suojanen (2022, 35) defines subjective safety as a feeling of whether a person feels threatened or not (see also, Suojanen and Thin, 2021; Suojanen et al., 2019). The emergence of sense of safety is influenced by how the environment and safety conditions meet the individual's safety needs (Zou and Meng, 2019).

Interdisciplinary research specifically on sense of safety is scarce and often lacks clear definitions of the concepts (as in, e.g., Zacharia and Yablon, 2022; Murakami et al., 2017) or fails to adequately address the relationship between safety and sense of safety (e.g., in Zou and Yu, 2022). Research and mainstream media focus mostly on the broader aspects of safety, including statistics, numerical trends, and political reporting. This Research Topic aims to provide deeper and more versatile insights by employing various methodological approaches (e.g., developing tools for in-depth qualitative interviews and ethnographic studies in the work of Pessi et al., as well as large-scale surveys and experimental materials in the studies conducted by Ren et al. and Cai et al.).

Many definitions and studies on sense of safety, as well as safety, focus on experienced threat or fear or the lack of it. However, positive aspects and the absence of concerns about safety have also been shown to be relevant for sense of safety (Brands et al., 2021). Indeed, human existence is never only about some potential and hypothetical threats and risks. Therefore, sense of safety must be based on something other than the absolute lack of threats—both research-wise and in everyday life.

This Research Topic explores how sense of safety operates through interconnected layers spanning from individual embodied experiences to broader societal structures. Taken together, these studies suggest a view of organizations and safety that embraces holistic perspectives, offering new insights beyond those available through a sole focus on either the physical or psychological aspects of safety. An example of this integrated perspective in this Research Topic can be seen in combining Lynch et al.'s “whole person experience” concept with Oertel's “corpomateriality” concept, Härkönen's work on communal spaces fostering belonging for minorities, and Pessi et al.'s spiral model highlighting social factors in emotionality—a blend that offers a perspective on sense of safety as manifesting through bodily sensations in resonance with and interaction against physical and relational spaces.

## The individual: sense of safety at the apex of individuals and the social

This Research Topic illustrates how sense of safety emerges from the interconnections between the individual and the shared. Sense of safety is about sensing. Therefore, a key element in all definitions of the concept is the individual's internal experience of safety (Zou and Meng, 2019; Nilsen et al., 2004). This individual experience is affected by internal (e.g., memories, past experiences), material, social and communal, societal, and symbolic and epistemic factors (e.g., Flyvholm and Johansen; Lynch et al.), all of which are intertwined. For example, although safety can be defined as the condition of being safe from hurt, injury, or loss (Merriam-Webster Dictionary, 2025)^1^, a person may feel unsafe without any visible threat or feel safe despite the presence of one.

Therefore, sense of safety is a subjective experience that is not always in line with observable safety conditions. As sense of safety is affected, among other things, by internal processes, which can cause a chronic state of unsafety, a person can feel unsafe even in the absence of overt stressors (Brosschot et al., 2018). The embodied processes of sensing safety or unsafety do not differentiate between objective and subjective threats (Fleshner and Laudenslager, 2004; Lynch et al.). Lynch et al. suggest that sense of safety is affected by one's life story, relationships, meaning, sense of self, and physical threat.

In psychotherapy, professionals constantly work with issues of safety, sense of safety, security, and insecurity. Cognitive reassurances of safety do not reach a fearful person. Rationality is, in a sense, unavailable when emotions and a heightened state of fear take over. The sense of safety experienced by an individual is largely shaped by what is and has been felt in connection with others. Particularly in early interactions, internalized experiences of others' safety and their ability to stay calm in the face of perceived danger are crucial. Our attention to others sharpens in the face of threat: Am I safe with the other person? Do I receive acceptance and opportunities to calm down under their protection? Experiences of internal insecurity can gradually transform into feelings of security and safety within a long term, trusting therapeutic relationship, perhaps for the first time in life. Sense of safety experienced together gradually internalizes, significantly impacting daily life.

This Research Topic, indeed, challenges the traditional divide between objective safety measures and subjective perceptions. For instance, Heino et al.'s work on “false sense of safety” highlights the dangers of relying exclusively on either objective metrics or subjective feelings. Three implications for practice emerge from challenging this divide. First, there is a need for multisensorial approaches to safety assessment, moving beyond an exclusive reliance on cognitive understanding. Second, there is a necessity for demographically informed interventions acknowledging how safety is experienced differently across groups and incorporating views on power. Third, there is a need to adopt systems thinking approaches to safety management that consider non-linear risks, tipping points, and the understanding of safety as dynamic rather than static (Wei et al.; Heino et al.; Wolff and Larsen).

## The relational: sense of safety in human relations and society

This Research Topic moves beyond fragmented approaches to safety and toward more comprehensive perspectives that view sense of safety as a complex phenomenon intertwined with and shaped by social relationships and organizational structures. Our Research Topic also highlights the importance of relational foundations for sense of safety, as seen in Zhao and Li's study on resilience and in Miralles et al.'s demonstration of how servant leadership creates psychological safety, in part through compassion. Compassion emerges as a key human experience warranting further exploration in relation to sense of safety throughout the Research Topic (e.g., Halamová et al.; Yin et al.; Lynch et al.).

Indeed, organizations are sites of power, and multiple studies have explored how sense of safety is crucially shaped by power dynamics. Rabelo et al. address how safety frameworks reflect broader societal power dynamics. This aligns with the findings of Cai et al., which indicate that employee groups experience safety differ partly based on their relative power positions. Pauha's hypothesis and theory article on real-contact stick fighting suggests that experiences of safety and attraction to danger or even threats to one's life and wellbeing are gendered and culturally structured. Various cultural contexts and their differences must indeed be carefully considered.

Shifting from public discourses to everyday interactions, it becomes clear that being in a minoritized position often makes people disproportionately vulnerable to experiences of discrimination and social exclusion. This may profoundly shape feelings of safety on various levels, and the article by Flyvholm and Johansen investigates a particular domain of safety concerns that emerges in everyday encounters for people in minoritized positions, namely safety concerns related to *epistemic* encounters. By tracing how Danish Muslims handle “difficult knowledge” about racism and hate victimization, the article reveals the delicate ways people try to balance different and often contradictory safety needs of staying informed, staying at ease, staying credible, staying truthful, and, through it all, staying hopeful.

Sense of safety is not equally distributed across society and communities. Indeed, how safe a person feels in different situations is likely to be predicated upon a variety of factors, such as identity traits (e.g., gender, religiosity, sexual, or racial identity), the places they move through (e.g., places of work, residence, and transportation), and prior experiences of unsafe situations (e.g., threats, harassment, or even violence). This invites us to explore the interconnectedness between embodied experiences of safety (or lack thereof) and the structural and political conditions on which these embodied experiences are predicated. For instance, in their contribution to this Research Topic, Rabelo et al. address the role of public discourses in distributing senses of safety, showing how ethnicity, power, and privilege shape people's sense of safety and danger. Through an analysis of three public safety frameworks, all driven by carceral logics, the article shows how these frameworks elevate the safety concerns of dominant groups while criminalizing undesirable bodies, undermining stigmatized communities' ability to access public safety and justice, and legitimizing suspicion and surveillance.

## The global: vistas for promoting sense of safety

Even more broadly, sense of safety is also experienced in a global context. Globally, the world not only feels fragile—it is, in fact, a fragile place facing a triple planetary crisis of climate change, biodiversity loss, and pollution. These environmental challenges are exacerbating a triple health crisis of inequitable access to quality healthcare, increasing health-related emergencies, and declining global health and wellbeing metrics. These, in turn, are both driving and being driven by a triple societal crisis of social erosion, which is aggravated by conflict and its associated systemic breakdowns; global financial insecurities, which is increasing the inequality gap between rich and poor; and the strange indivisibility of truth and mistruth, leading to the destruction of trust.

However, the global is experienced through the individual. For example, Ferretti et al., in examining the relationship between feelings of urban safety, note that individual characteristics and experiences play the most significant role—even amidst global crises such as the COVID-19 pandemic.

In conditions of fragility, there exist levers for change. The articles in this review highlight some of the global and local levers, showing that the foundation of support for individuals and systems lies in a shared belief in commonality practiced through compassion. The strength of this review Research Topic lies in its core message: knowledge generated in one context can resonate across others, opening up new ways of being in the world and radically transforming perceived obstacles to human flourishing.

The spiral model (Pessi et al.), developed to explore the sense of safety experienced in the spaces of religion, offers unique insights into how to understand and foster a communal sense of safety. The very act of searching for and articulating this sense of safety is, in itself, a revolutionary endeavor. This review Research Topic invites us to ponder whether—and how—we might find the collective courage to bring about the change that our world needs.

Revolutions occur when, in the very midst of places and spaces where safety has been diminished or destroyed, a small but communal sense of safety emerges, giving strength to those who see the world differently and who know that the future can be reimagined.

Accepted and familiar geopolitical securities are shifting rapidly, while new narratives have yet to catch up and fill the resulting gaps. The hope and shared compassion that inspired 193 nations to collectively sign the Sustainable Development Goals appear to be ebbing away as countries turn inward to strengthen their national identities. The collective belief underpinning the global securitization agenda—that the SDGs were not only beneficial for people, the planet, and prosperity but also essential for peace—has disappeared.

We can continuously nurture each other's sense of safety through small, everyday actions. These small deeds spread across social networks, building collective feelings of safety, manifesting, for example, in the efforts of NGOs and grassroots activism. Ultimately, societal safety and security are deeply rooted in these everyday acts. For sense of safety, small is all.

## Footnotes

Edited and reviewed by: Gerald Matthews, George Mason University, United States
